# The role of microbiota in compensatory growth of protein‐restricted rats

**DOI:** 10.1111/1751-7915.12451

**Published:** 2016-11-22

**Authors:** Yizhi Zhu, Qingyan Niu, Chao Shi, Jing Wang, Weiyun Zhu

**Affiliations:** ^1^ Jiangsu Key Laboratory of Gastrointestinal Nutrition and Animal HealthLaboratory of Gastrointestinal MicrobiologyCollege of Animal Science and TechnologyNanjing Agricultural UniversityNanjing210095China

## Abstract

Compensatory growth is a physiological phenomenon found in both humans and animals. However, the underlying mechanisms are unclear. In this study, for the first time, we investigated the role of microbiota in compensatory growth induced by protein restriction using a rat model. Weaned Sprague‐Dawley rats were fed a low protein diet (L group), a normal protein diet (N group) and a low protein diet for 2 weeks followed by a normal protein diet (LN group). The results showed that in contrast with the inhibited growth of rats in the L group, compensatory growth was observed in the LN group. Meanwhile, rats in the LN group had increased concentrations of total short chain fatty acids, particularly butyrate, and an altered bacterial composition with modified abundances of Peptostreptococcaceae, Bifidobacteriaceae, Porphyromonadaceae and Prevotellaceae in the colonic content. Furthermore, gene expression analysis indicated that the rats that experienced compensatory growth had improved barrier function and innate immune function in the colon. Our data revealed the importance of colonic microbiota in achieving compensatory growth.

## Introduction

Low‐birth weight neonates and malnourished children usually exhibit compensatory growth due to foetal malnutrition or postnatal protein/calorie malnutrition (Ashworth, 1969; Hack *et al*., 1996). Compensatory growth is defined as the physiological phenomenon of accelerated growth rate in organisms after restricted nutrition or food intake (Hornick *et al*., 2000). According to the previous studies, the changes in circulating hormones (including growth hormone, insulin growth factor I and insulin) as well as the metabolic changes in multiple organs and tissues (including liver, pancreas, intestine, muscle and fat tissue) are believed to be causal factors or consequences of compensatory growth (Wilson and Osbourn, 1960; Hornick *et al*., 2000). Among these organs and tissues, the gastrointestinal tract (GIT) is crucial for host metabolism and health, because it fulfils the nutritional requirements for the whole host and is the largest endocrine and immune organ in the body (Borgstrom *et al*., 1979; Evans *et al*., 2013; Peterson and Artis, 2014). However, the underlying mechanisms of GIT involvement in compensatory growth are still unclear. Thus, further investigations focusing on GIT are needed to elucidate its role in compensatory growth.

The GIT contains a complex mixture of compounds from alimentary and endogenous origins and a number of living microorganisms (Hamer *et al*., 2012).Specifically, the large intestine harbours the most dense and metabolically active microbial community (> 10^11^ cells per gram content), which is an important environmental factor that contributes to host metabolism (Eckburg *et al*., 2005; Bäckhed, 2011). Bäckhed *et al*. (2004) demonstrated that mice colonized with normal microbiota gained significantly more body fat than germ‐free mice, although they had a lower feed intake. Other studies showed that transplantation of microbiota from obese mice to germ‐free mice resulted in obesity in the recipient mice, while they stayed lean when the microbiota were transplanted from lean mice (Turnbaugh *et al*., 2006; Ridaura *et al*., 2013). Gut microbiota can modulate gene expression in the colonic mucosa and contribute to host metabolic efficiency through promotion of energy storage by increasing energy availability via production of short chain fatty acids (SCFAs) in the colon (Bäckhed *et al*., 2004, 2005; Wichmann *et al*., 2013). Therefore, in this study, we hypothesized that the microbes in the colon play a crucial role in compensatory growth. To verify our hypothesis, we constructed a compensatory growth model induced by protein restriction using weaned rats, and investigated the changes in microbial composition and metabolism in the colon as well as the expression of genes related to gut function. Although recent studies in both rats and piglets have shown that a low protein (LP) diet or protein restriction altered the microbial composition in colon such as decreased lactobacilli (Rist *et al*., 2013) and prevented body weight gain (Maia *et al*., 2014), studies examining the effect of protein realimentation on colonic microbiota and subsequent growth are scarce. Therefore, our study provides evidence that colonic microbiota facilitated host compensatory growth after protein realimentation.

## Results

### Growth performance

**Figure 1:**
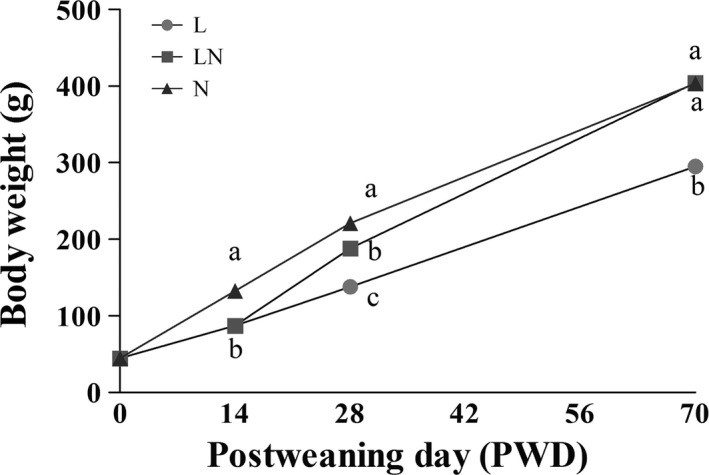
The average body weight of the rats in each group on PWD 14, PWD 28 and PWD 70. During the period from PWD 0 to PWD 14, rats were either fed a low protein diet (L group, 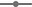, *n* = 6) or a normal protein diet (N group, 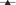, *n* = 6). After PWD 14, 12 rats from the L group were switched to the normal protein diet with two rats housed in a cage to induce compensatory growth (LN group, 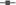, *n* = 6). Different letters among groups on the same day indicate a significant difference, *P* < 0.05.

During the whole experimental period, the body weight gain of rats in the L group was significantly inhibited compared with the N group (Fig. 1). However, a compensatory increase in body weight was observed on post‐weaning day (PWD) 28 in the LN group after rats were switched to a normal protein (NP) diet on PWD 14, although the value was still lower than that in the N group. Furthermore, the body weight of rats in the LN group did not differ from that in the N group on PWD 70 (Fig. 1), indicating compensatory growth. Accordingly, rats in the L group had the lowest average daily gain (ADG), average daily feed intake (ADFI) and feed conversion rate compared with those in the LN and N groups throughout the experiment (Table 1). However, ADG was unaffected and ADFI was decreased in the LN group compared with the N group from PWD 14 to PWD 28. Therefore, rats in the LN group had the highest feed conversion rate. During the period from PWD 28 to PWD 70, the ADFI in the LN group did not differ from that in the N group, while the ADG and feed conversion rate were higher in the LN group than those in the N group.

**Table 1:** The average daily gain (ADG, g day^−1^), average daily feed intake (ADFI, g day^−1^) and Feed/Gain (F/G) ratio during the experimental periods

Item	L	LN	*N*	SEM	*P*‐value
PWD (0–14)					
ADG	3.04^a^		6.28^b^	0.13	<0.001
ADFI	8.27		8.84	0.16	0.05
F:G ratio	2.74^a^		1.41^b^	0.05	<0.001
PWD (14–28)					
ADG	3.88^a^	7.38^b^	7.21^b^	0.19	<0.001
ADFI	11.84^a^	11.95^a^	17.58^b^	0.47	<0.001
F:G ratio	3.06^a^	1.63^b^	2.44^c^	0.09	<0.001
PWD (28–70)					
ADG	3.75^a^	5.15^b^	4.35^c^	0.12	<0.001
ADFI	14.12^a^	16.55^b^	18.07^b^	0.55	0.001
F/G ratio	3.79^a^	3.22^b^	4.16^a^	0.14	0.001

During the period from PWD 0 to PWD 14, rats were either fed a low protein diet (L group, *n* = 6) or a normal protein diet (N group, *n* = 6). After PWD 14, 12 rats from the L group were switched to the normal protein diet with two rats housed in a cage to induce compensatory growth, as the LN group (*n* = 6). Means in a same row with different superscripts indicate a significant difference (*P* < 0.05).

### Bacterial metabolites in the colonic content

To assess the microbial metabolism in the colon, the SCFAs and ammonia‐N in the colonic content were measured. The concentration of total SCFAs in the colonic content of rats in the L group was decreased compared with the N group on PWD 28 and PWD 70 with no significant statistical difference on PWD 14 (Table 2). However, the concentration of total SCFAs in the LN group on PWD 28 was higher than that in the N group, with no significant difference on PWD 70. Specifically, the propionate concentration was decreased in the L group on PWD 14, 28 and 70 compared with the N group (Table 2). Shifting to the NP diet restored the propionate concentration on PWD 28 in the LN group, but not on PWD 70. In addition, a decreased concentration of acetate was observed on PWD 70, but not on PWD 28 in the L group. The acetate concentration in the LN group did not differ from that in the N group on both PWD 28 and PWD 70. Meanwhile, no difference was detected at the concentrations of butyrate and isobutyrate between the L and N groups on PWD 28 and PWD 70 respectively. However, elevated concentrations of butyrate and isobutyrate were observed in the LN group on PWD 28. As shown in Table 2, the ammonia‐N concentration in the colon was not significantly affected by the dietary treatments.

**Table 2:** The concentration of SCFAs (μmol g^−1^ content) and ammonia‐N (mmol g^−1^ content) in the colonic content

Item	L	LN	*N*	SEM	*P*‐value
PWD 14					
Acetate	61.82		54.57	3.19	0.17
Propionate	7.81^a^		9.41^b^	0.38	0.02
Isobutyrate	0.78		0.76	0.06	0.87
Butyrate	16.27		12.86	1.90	0.23
Isovalerate	0.72		0.86	0.06	0.13
Valerate	0.99		0.57	0.14	0.06
Total SCFA	88.39		79.01	3.94	0.14
Ammonia‐N	1.11		1.12	0.09	0.94
PWD 28					
Acetate	35.54^a^	49.57^b^	41.62^ab^	3.29	0.04
Propionate	3.78^a^	9.43^b^	11.49^b^	0.84	0.00
Isobutyrate	0.45^a^	0.74^b^	0.44^a^	0.07	0.01
Butyrate	11.13^a^	23.38^b^	10.38^a^	0.77	0.00
Isovalerate	0.39	0.72	0.72	0.13	0.16
Valerate	0.35^a^	1.07^ab^	1.28^b^	0.23	0.06
Total SCFA	51.63^a^	84.91^b^	65.93^c^	4.30	0.00
Ammonia‐N	0.73	0.94	0.92	0.10	0.28
PWD 70					
Acetate	40.78^a^	46.98^ab^	48.82^b^	2.29	0.07
Propionate	6.67^a^	8.96^b^	10.87^c^	0.55	0.00
Isobutyrate	0.77	0.82	0.74	0.09	0.83
Butyrate	9.50	12.06	11.07	1.14	0.33
Isovalerate	0.95	0.88	0.81	0.08	0.47
Valerate	0.94	0.78	0.89	0.07	0.31
Total SCFA	59.60^a^	70.48^b^	73.19^b^	3.17	0.02
Ammonia‐N	0.64^a^	1.06^b^	0.74^ab^	5.72	0.06

During the period from PWD 0 to PWD 14, rats were either fed a low protein diet (L group, *n* = 6) or a normal protein diet (N group, *n* = 6). After PWD 14, 12 rats from the L group were switched to the normal protein diet with 2 rats housed in a cage to induce compensatory growth, as the LN group (*n* = 6). Means in the same row with different superscripts indicate a significant difference (*P* < 0.05).

### Data acquisition by MiSeq sequencing and the bacterial diversity in the colonic content

In this study, the number of average raw sequences detected in a group was at least 19 391 reads, with 15 386 valid sequences (Table 3). The overall number of OTUs detected was 5647, based on a 97% sequence similarity between reads. The sampling was sufficient to evaluate the bacterial community profile as shown by the rarefaction curves and the species accumulation curve (Fig. S1). As shown in Table S1, the bacterial diversity in the colonic content of rats in the L group decreased on PWD 28. For the colonic bacterial diversity of rats in the LN group, the values of observed OTUs, Shannon, Simpson and Chao 1 indices did not differ from those in the N group on PWD 28 and PWD 70, which indicated that protein restriction for 14 days did not affect the bacterial diversity in the colonic content of rats in the LN group.

**Table 3:** The average clean data acquired during sequencing

Groups	Raw data	Valid data	Valid%	Q20%	Q30%	GC%
PWD 14						
L	22 322.00	17 933.00	80.36	91.77	78.19	52.76
N	22 733.00	17 822.67	78.19	90.62	76.07	53.46
PWD 28						
L	19 391.33	15 386.00	79.39	91.21	77.26	52.28
LN	29 284.00	23 619.67	80.67	91.99	78.51	53.40
N	20 399.67	16 385.17	80.28	91.16	77.22	53.53
PWD 70						
L	21 792.17	17 003.17	77.92	90.90	76.67	52.84
LN	23 810.67	18 861.67	79.15	91.19	77.17	53.18
N	21 981.17	17 389.50	79.12	90.92	76.91	53.34

The number of average raw sequences detected in a group was at least 19 391 reads, with 15 386 sequences. The indexes of quality control Q20 and Q30 were sufficient to ensure the accuracy of sequencing.

### Bacterial composition in the colonic content determined by MiSeq sequencing

**Figure 2:**
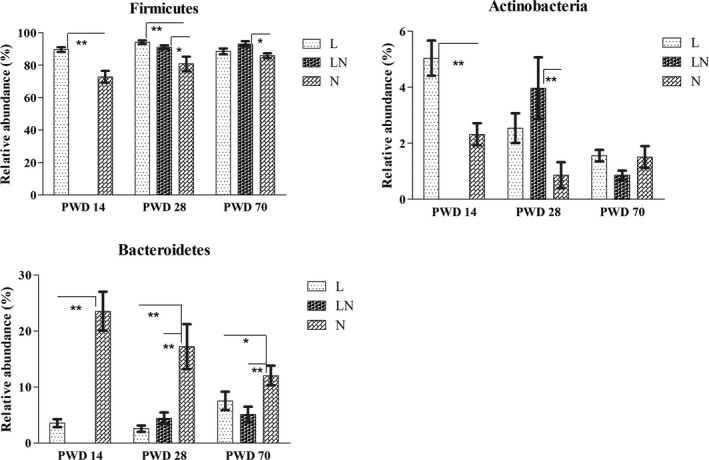
Comparisons of the average relative abundances of Firmicutes, Actinobacteria and Bacteroidetes in colonic content of each group on PWD 14, PWD 28 and PWD 70. During the period from PWD 0 to PWD 14, rats were either fed a low protein diet (L group, 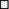, *n* = 6) or a normal protein diet (N group, 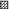, *n* = 6). After PWD 14, 12 rats from the L group were switched to the normal protein diet with 2 rats housed in a cage to induce compensatory growth (LN group, 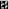, *n* = 6). Values are presented as the mean ± SEM. * indicates *P* < 0.05; ** indicates *P* < 0.01.

The bacterial composition was investigated at different taxonomic levels. At the phylum level, the most dominant group was Firmicutes, accounting for 72.90–94.18% of the total sequences (Fig. S2), followed by the bacteria from the phyla Bacteroidetes (2.58–23.54%), Actinobacteria (0.86–5.04%), Candidatus Saccharibacteria (0.12–1.36%) and Proteobacteria (0.25–0.92%). The rarely detected phyla including Tenericutes and Verrucomicrobia, which had an average relative abundance of < 0.1%, were not identified in the subsequent analysis. Statistically, the abundance of Firmicutes, Bacteroidetes and Actinobacteria was significantly affected by the dietary treatments (Fig. 2). The abundance of Firmicutes in the L group was increased by protein restriction on PWD 14 and PWD 28 compared with the N group. In addition, an elevated abundance of Firmicutes was also observed on PWD 28 and PWD 70 in the LN group after the rats were switched to the NP diet (Fig. 2). Moreover, the abundance of Actinobacteria was increased in the L group on PWD 14 (Fig. 2) and an elevated abundance of Actinobacteria was observed in the LN group on PWD 28. However, the abundance of Bacteroidetes (Fig. 2) was decreased in the L group on PWD 14, PWD 28 and PWD 70 and was consistently decreased on PWD 28 and PWD 70 in the LN group compared with that in the N group.

**Figure 3:**
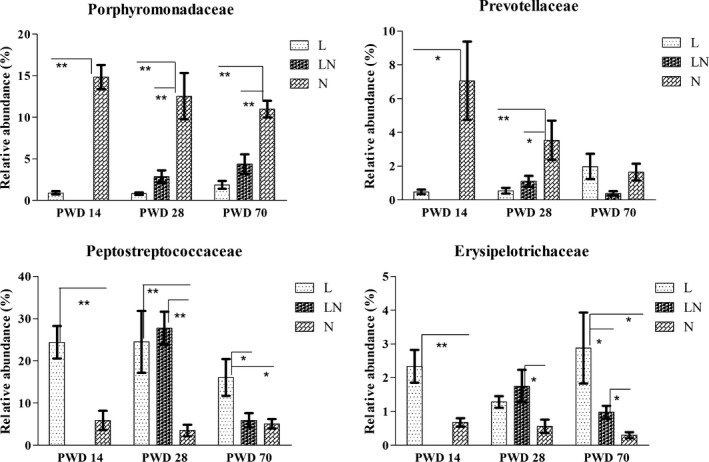
Comparisons of the average relative abundances of altered bacteria at the family level in colonic content of each group on PWD 14, PWD 28 and PWD 70. During the period from PWD 0 to PWD 14, rats were either fed a low protein diet (L group, 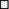, *n* = 6) or a normal protein diet (N group, 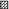, *n* = 6). After PWD 14, 12 rats from the L group were switched to the normal protein diet with two rats housed in a cage to induce compensatory growth (LN group, 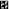, *n* = 6). Values are presented as the mean ± SEM. * indicates *P* < 0.05; ** indicates *P* < 0.01.

At the family level, 52 families were detected, including six unclassified ones. The bacterial taxa that had an average relative abundance of > 1% in at least one group, were statistically analysed. As a result, 16 families of bacteria were assessed (Fig. S3), with Lactobacillaceae the most predominant (28.40–58.70%). The abundance of Porphyromonadaceae was significantly decreased on PWD 14, PWD 28 and PWD 70 in the L group compared with the N group (Fig. 3). Furthermore, a decreased abundance of Porphyromonadaceae was consistently observed in the LN group on PWD 28 and PWD 70. Similar results were found in the abundance of Prevotellaceae (Fig. 3), which was decreased in both the L and the LN groups on PWD 14 and PWD 28. However, the abundance of Peptostreptococcaceae was increased in the L group on PWD 14, PWD 28 and PWD 70 (Fig. 3) and persistently elevated on PWD 28 in the LN group compared with the N group. In addition, the abundance of Erysipelotrichaceae was increased in the L group on PWD 14 and PWD 70 (Fig. 3) and an increased abundance of Erysipelotrichaceae was consistently observed on PWD 28 and PWD 70 in the LN group.

**Figure 4:**
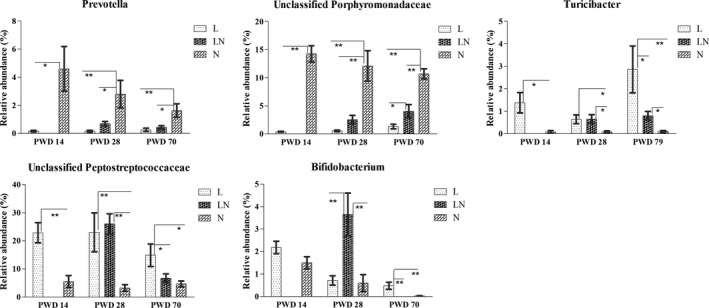
Comparisons of the average relative abundances of altered bacteria at the genus level in colonic content of each group on PWD 14, PWD 28 and PWD 70. During the period from PWD 0 to PWD 14, rats were either fed a low protein diet (L group, 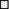, *n* = 6) or a normal protein diet (N group, 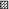, *n* = 6). After PWD 14, 12 rats from the L group were switched to the normal protein diet with two rats housed in a cage to induce compensatory growth (LN group, 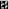, *n* = 6). Values are presented as the mean ± SEM. * indicates *P* < 0.05; ** indicates *P* < 0.01.

At the genus level, 103 genera, including 23 unclassified genera, were detected. The genera that had a relative abundance of > 2% in at least one group were analysed. As a result, 13 genera of bacteria, including the unclassified Porphyromonadaceae, Lachnospiraceae, Peptostreptococcaceae and Ruminococcaceae were analysed (Fig. S4). The most dominant genus was *Lactobacillus* (28.40–58.70%), followed by unclassified Lachnospiraceae (6.98–33.66%) and unclassified Peptostreptococcaceae (3.22–26.03%). The abundance of *Prevotella* and unclassified Porphyromonadaceae was decreased in the L group on PWD 14, PWD 28 and PWD 70 compared with the N group (Fig. 4). Furthermore, the abundance of *Prevotella* and unclassified Porphyromonadaceae was decreased in the LN group on PWD 28 and PWD 70. However, the abundance of *Turicibacter* and unclassified Peptostreptococcaceae was increased in the L group on PWD 14, PWD 28 and PWD 70 (Fig. 4). Moreover, the abundance of *Turicibacter* was increased in the LN group on PWD 28 and PWD 70. The abundance of unclassified Peptostreptococcaceae was increased on PWD 28 in the LN group. For *Bifidobacterium* (Fig. 4), a higher abundance of *Bifidobacterium* was also observed in the LN group on PWD 28 and the L group on PWD 70 compared with the N group.

### Quantification of the total bacteria in the colonic content by q‐PCR

**Figure 5:**
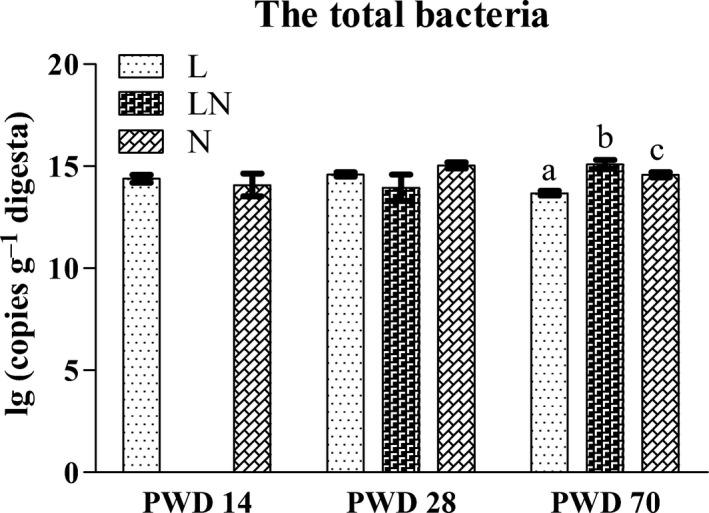
Gene copies of total bacteria in the colonic content on PWD 14, PWD 28 and PWD 70. During the period from PWD 0 to PWD 14, rats were either fed a low protein diet (L group, 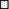, *n* = 6) or a normal protein diet (N group, 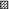, *n* = 6). After PWD 14, 12 rats from the L group were switched to the normal protein diet with 2 rats housed in a cage to induce compensatory growth (LN group, 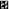, *n* = 6). Values are presented as the mean ± SEM. * indicates *P* < 0.05; ** indicates *P* < 0.01.

As MiSeq sequencing did not reveal the absolute number of total bacteria in the colonic content, the gene copies of total bacteria were quantified by q‐PCR. The results (Fig. 5) showed that the gene copies of total bacteria in the colonic content were not affected by protein restriction on PWD 14. In addition, no difference was observed in the L and LN groups on PWD 28 compared with the N group. A significantly decreased number of total bacteria was observed in the L group on PWD 70 (Fig. 5). However, the number of total bacteria in the LN group was increased on PWD 70 compared with the N group.

### Predicted molecular functions of the bacteria in the colonic content

**Figure 6:**
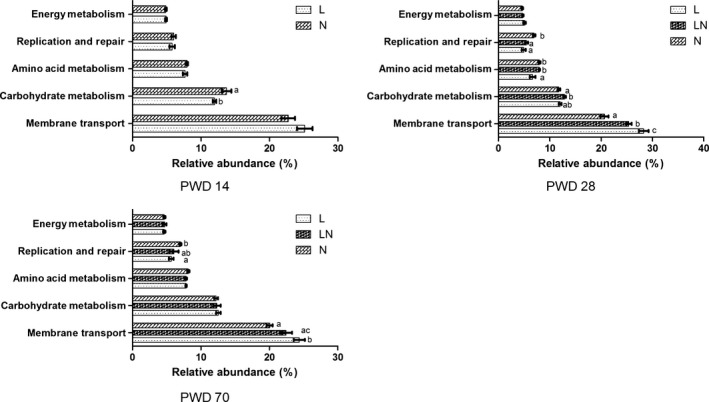
Comparisons of the top five major KEGG pathways of bacteria in the colonic content on PWD 14, PWD 28 and PWD 70. During the period from PWD 0 to PWD 14, rats were either fed a low protein diet (L group, 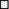, *n* = 6) or a normal protein diet (N group, 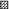, *n* = 6). After PWD 14, 12 rats from the L group were switched to the normal protein diet with 2 rats housed in a cage to induce compensatory growth (LN group, 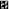, *n* = 6). Different letters among groups indicate a significant difference, *P* < 0.05.

To gain insight into the molecular functions of bacteria in the colonic content, the online galaxy version of PICRUSt was used to predict the metagenome contribution of the communities observed. In this study, 37 KEGG pathways were identified in the colonic content (Fig. S6). Among the 37 genes identified, the top five major KEGG pathways were membrane transport (14.39–32.43%), carbohydrate metabolism (10.87–16.83%), amino acid metabolism (4.39–8.84%), replication and repair (2.86–8.23%) and energy metabolism (4.02–6.09%). Significant differences were observed by further comparisons of the relative abundance of the top five KEGG pathways. The results showed that the relative abundance of carbohydrate metabolism was decreased in the L group on PWD 14 (Fig. 6) and increased in the LN group on PWD 28. The abundance of amino acid metabolism was decreased in the L group on PWD 28 (Fig. 6). In addition, a decreased abundance of replication and repair was observed in the L group on PWD 28 and PWD 70, and the same result was observed in the LN group on PWD 28 (Fig. 6), compared with the N group. Meanwhile, the abundance of membrane transport was increased in the LN group on PWD 28 and the L group on PWD 28 and PWD 70.

### Gene expressions in the colonic mucosa

**Figure 7:**
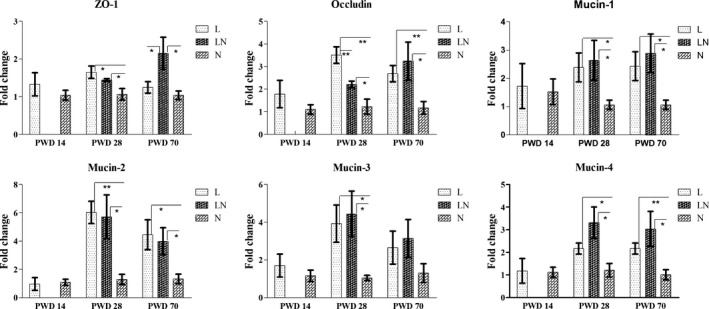
Expression of genes related to colonic barrier function including tight junction proteins and mucins on PWD 14, PWD 28 and PWD 70. During the period from PWD 0 to PWD 14, rats were either fed a low protein diet (L group, 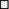, *n* = 6) or a normal protein diet (N group, 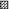, *n* = 6). After PWD 14, 12 rats from the L group were switched to the normal protein diet with two rats housed in a cage to induce compensatory growth (LN group, 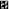, *n* = 6). Values are presented as the mean ± SEM. * indicates *P* < 0.05; ** indicates *P* < 0.01.

**Figure 8:**
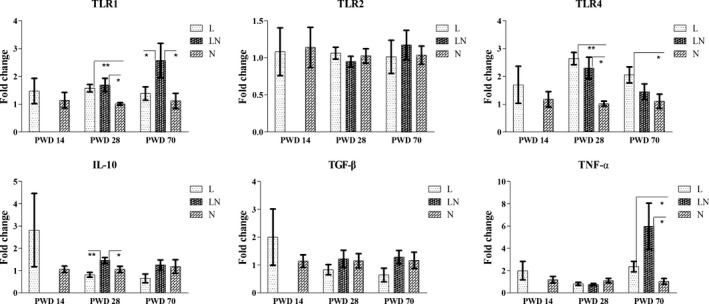
The relative expression of Toll‐like receptors (TLR1, TLR2 and TLR4) and inflammatory cytokines (IL‐10, TGF‐β and TNF‐α) on PWD 14, PWD 28 and PWD 70. During the period from PWD 0 to PWD 14, rats were either fed a low protein diet (L group, 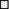, *n* = 6) or a normal protein diet (N group, 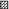, *n* = 6). After PWD 14, 12 rats from the L group were switched to the normal protein diet with two rats housed in a cage to induce compensatory growth (LN group, 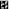, *n* = 6). Values are presented as the mean ± SEM. * indicates *P* < 0.05; ** indicates *P* < 0.01.

To assess the gene expression related to gut function, several genes related to gut barrier function and innate immune function were investigated by q‐PCR. The results showed that restricting the dietary protein of rats for 14 days in the L group did not affect the gene expression of tight junction proteins (ZO‐1 and occludin) compared with the N group (Fig. 7). However, the gene expression of ZO‐1 and occludin was increased on PWD 28 in the L and LN groups. On PWD 70, ZO‐1 and occludin both showed higher expression in the LN group compared with the N group. Meanwhile, the gene expression of occludin was also increased in the L group. For the gene expression of mucins (Fig. 7), protein restriction did not affect the expression of mucin‐1, mucin‐2, mucin‐3 and mucin‐4 on PWD 14. However, these genes were all significantly increased in the L and LN groups on PWD 28 compared with the N group. On PWD 70, increased gene expression of mucin‐1, mucin‐2 and mucin‐4 was observed in the L and LN groups, although gene expression of mucin‐3 was unchanged. For the genes related to gut innate immune function, the Toll‐like receptors (TLRs) and inflammatory cytokines were assayed. Figure 8 shows that the TLR1 and TLR4 had higher expression in the L and LN groups on PWD 28 compared with the N group. On PWD 70, the gene expression of TLR1 was only increased in the LN group and TLR4 was only increased in the L group. However, the gene expression of TLR2 did not differ among the groups. For the inflammatory cytokines (Fig. 8), the gene expression of transforming growth factor‐β (TGF‐β) remained unaffected throughout the experiment. However, the expression of interleukin‐10 (IL‐10) was increased in the LN group on PWD 28 and the expression of tumour necrosis factor‐α (TNF‐α) was increased in the L and LN groups on PWD 70 compared with the N group.

## Discussion

Dietary protein is essential for muscle growth and the synthesis of bioactive compounds such as hormones and neurotransmitters, both of which are crucial for growth and development. Hence, the body weight gain of rats was inhibited by protein restriction in the L group, which was consistent with the findings of Passos *et al*. (2000). However, significant compensatory growth was observed in the LN group, resulting in a similar body weight on PWD 70 compared with the N group. These results were consistent with previous findings on compensatory growth in rats (Horie and Ashida, 1971; Miyazawa and Kametaka, 1984; Ishida *et al*., 2011). The increased feed conversion rate in the LN group was one of the direct factors leading to the compensatory growth. However, the mechanisms require further investigation.

In our study, we focused on the role of colonic microbes in compensatory growth. Therefore, we assayed the composition and concentrations of SCFAs in colonic content by gas chromatography (GC) and used a MiSeq sequencing platform to determine the changes in bacterial composition in the colonic content of rats in the L and LN groups. Our results were consistent with previous findings that Firmicutes (principally Lactobacillaceae, Peptostreptococcaceae, Lachnospiraceae and Erysipelotrichaceae), Bacteroidetes (principally Porphyromonadaceae and Prevotellaceae), Actinobacteria (principally Bifidobacteriaceae, Coriobacteriaceae and Corynebacteriaceae) and Proteobacteria (principally Helicobacteraceae) were the predominant bacteria in the colon (Claus *et al*., 2011; Lin *et al*., 2016).

The gut microbiota could enhance the host metabolic capacity to process nutrients and promote energy harvest by fermenting carbohydrates into short‐chain fatty acids (Bäckhed *et al*., 2004; Ramakrishna, 2013). Moreover, investigations found that diet, particularly dietary protein, was the major factor that influenced the composition of the microbiota (Rist *et al*., 2013). Therefore, we further compared the concentrations of SCFAs and the relative abundance of bacteria at different taxonomic levels in the colonic content of rats in different groups. The increased concentration of total SCFAs (principally butyrate and isobutyrate) in the LN group on PWD 28 indicated a higher amount of energy extraction from the colonic content. Bäckhed *et al*. (2004) and Samuel *et al*. (2008) reported that the altered colonic microbial composition and the increased concentrations of SCFAs could promote liver lipogenesis and adiposity. Thus, it is reasonable to speculate that the increased SCFA concentration in rat colons facilitated the compensatory growth in the LN group. First of all, altered bacterial composition can lead to the increased total SCFA concentration. The increased gene copies of total bacteria in the LN group on PWD 70 suggested that the total fermentative capacity of bacteria in the LN group was increased, promoting increased production of SCFAs. In addition, the enriched abundance of Firmicutes‐Peptostreptococcaceae and Actinobacteria‐ Bifidobacteriaceae‐ *Bifidobacterium* in the LN group was also responsible for the increased amount of total SCFAs, as they were shown to be associated with increased energy intake (Jumpertz *et al*., 2011) and butyrate production (Macfarlane *et al*., 2013; Lin *et al*., 2016). Moreover, increased abundance of KEGG pathways in carbohydrate metabolism in the LN group was observed on PWD 28, indicating that the ability of bacteria to degrade carbohydrates was improved. This increase resulted in increased SCFA production. Second, the SCFAs could be derived from both carbohydrates and proteinaceous substrates (Puertollano *et al*., 2014). The increased isobutyrate in the colonic content of rats in the LN group indicated greater protein fermentation, as this compound is exclusively produced by protein degradation (Zhou *et al*., 2016). After the rats were switched to the NP diet, a higher proportion of undigested protein reached to the colon, resulting in greater protein fermentation and increased production of SCFAs. As the most affected SCFA, butyrate is one of the preferred energy sources for the epithelial cells in the colon and can be used as a substrate for lipogenesis (Carmona and Freedland, 1989; Butzner *et al*., 1996). The nearly doubled concentration of butyrate in the LN group facilitated the compensatory growth. Therefore, the altered bacterial composition and the increased SCFAs (particularly butyrate) led to the increased efficiency of energy absorption in the colon, which further contributed to the compensatory growth of rats in the LN group (Ramakrishna, 2013). In contrast with the LN group, the decreased concentrations of SCFAs and altered bacteria in colonic content of rats in the L group were consistent with the poor growth performance. The decreased gene copies of total bacteria on PWD 70 and the decreased abundance of KEGG pathways in amino acid metabolism on PWD 28 in the L group both indicated poor SCFA production capacity. Moreover, the decreased abundance of *Prevotella* (OTU 1960) and *Bacteroides* (OTU 1802), as shown in Figure S5, may also contribute to the decreased SCFA production as they were both associated with the production of SCFAs (De Filippo *et al*., 2010; Ramakrishna, 2013).

The interactions between the gut microbiota and host cells regulate gut immune function and gene expression as well as host metabolism (Hooper *et al*., 2012; Tremaroli and Bäckhed, 2012). Altered microbial composition and metabolism in the colon would also impact gut gene expression. Therefore, the expression of genes related to colonic function was also investigated. The up‐regulated expression of ZO‐1, occludin and mucins in the LN group on PWD 28 and PWD 70 indicated improved barrier function. Previous studies demonstrated that SCFAs (particularly butyrate) were capable of maintaining barrier function by promoting the expression of mucins, ZO‐1 and occludin (Finnie *et al*., 1995; Willemsen *et al*., 2003; Peng *et al*., 2007). Thus, the increased concentrations of SCFAs in the LN group contributed to the up‐regulated expression of ZO‐1, occludin and mucins. In addition, *Bifidobacterium* was shown to enhance the expression of ZO‐1 and occludin (Ewaschuk *et al*., 2008; Chichlowski *et al*., 2012). Moreover, several strains of *Bifidobacterium* were also shown to degrade mucins (Ruas‐Madiedo *et al*., 2008). The degradation of mucins by *Bifidobacterium* may induce the transcription of mucin genes. Therefore, the increased abundance of Bifidobacteriaceae in the LN group was associated with the upregulation of these genes. The Toll‐like receptors (TLR1, TLR2, TLR4) are key mediators of the innate host defense in the intestinal mucosa and are involved in maintaining mucosal as well as commensal homeostasis. TLR signalling in a healthy body protects epithelial barrier function and confers commensal tolerance. When pathogens invade, TLR signalling stimulates diverse inflammatory responses, leading to the secretion of pro‐inflammatory/anti‐inflammatory cytokines (Cario, 2005). Therefore, the up‐regulated TLRs in the LN group indicated improved innate immune function. The increased expressions of TLR1 and TLR4 were also associated with the elevated expression of IL‐10 on PWD 28 and TNF‐α on PWD 70 in the LN group. In addition, the increased abundance of Bifidobacteriaceae again contributed to the increased expression of IL‐10 as Tanabe *et al*. (2008) reported that bifidobacteria induced intestinal IL‐10 production. In general, the colonic barrier and innate immune functions were both improved in the LN group, which was essential to ensure the compensatory growth of rats.

In this study, we aimed to investigate the role of colonic microbiota in compensatory growth and the most striking result was the increased butyrate production in the colon. Whether the increased butyrate in the colon promotes compensatory growth is currently under investigation. In addition, the contribution of GIT to compensatory growth is not limited to the colon. We consider that the upper GIT, such as jejunum and ileum, also participates in this process, which will be investigated in our future studies.

In conclusion, the compensatory growth of rats, induced by protein restriction, was accompanied by altered bacterial composition in the colon. The altered bacterial composition resulted in a modified metabolic phenotype with increased production of SCFAs, particularly butyrate. Simultaneously, the expressions of genes related to barrier function and innate immune function in colon were increased. These results are partially responsible for the compensatory growth of rats.

## Experimental procedures

### Animal trial

The animal experiments were approved by the Animal Experiment Committee of Nanjing Agriculture University, in accordance with the Regulations for the Administrations of Affairs Concerning the Experimental Animals (The State Science and Technology Commission of China, 1988). All experiments were performed in accordance with the approved guidelines and regulations.

Forty‐eight Sprague‐Dawley male rats with an initial average body weight of 44.7 ± 1.51 g, weaned at 21 days of age, were used in the study. Eighteen rats were randomly selected, divided into six cages, and fed a NP diet as the control group (N group), with three rats housed in the same cage. The remaining 30 rats (L group) were randomly allocated into six cages and fed a LP diet until PWD 14, with five rats in each cage. Diets were formulated according to the AIN‐93G formula designated for the growth, pregnancy and lactational phases of rodents (Reeves *et al*., 1993) (Table S2). On PWD 14, six rats from the N group and six rats from the group were sacrificed for sampling. On the same day (PWD14), rats from the L group were mixed and then randomly reassigned to an L group (rats were continuously fed with the LP diet) and an LN group (rats were fed with the NP diet). To exclude the mixing effect in the L and LN groups, rats in the N group were also mixed and reassigned to six cages, with two rats in each cage. On PWD 28 and PWD 70, six rats from each group were sacrificed for sampling.

During the whole experimental period, rats were raised at a constant temperature (25°C) and humidity (70%) on a 12 h light/dark cycle. Water and food were fed *ad libitum* throughout the experiment. The feed intake and body weight were recorded to calculate the ADFI and ADG. Blood was sampled before the rats were sacrificed. The abdomen was opened and the whole GIT was removed. Colonic content was sampled and immediately stored at −20°C for later bacterial DNA and metabolite analyses. Mucosa was collected by scraping with slides and immediately stored in liquid nitrogen for later RNA extraction.

### Determination of microbial metabolites

The ammonia‐N in the colonic content was determined using the method described by Weatherburn (Weatherburn, 1967). For the determination of SCFAs, GC (Agilent 7890A, Agilent, Palo Alto, CA, USA) was used with a Nukol™ Capillary GC Column (Sigma‐Aldrich, Bellefonte, Pennsylvania, USA) and a FID detector as described by Mao *et al*. (2008). The temperatures of the injector, column and detector were 110°C, 135°C and 180°C respectively.

### Illumina MiSeq sequencing and data processing

The bacterial DNA was extracted using a bead‐beating method with a mini‐bead beater (Biospec Products, Bartlesville, OK, USA), followed by phenol‐chloroform extraction (Zoetendal *et al*., 1998). The solution was precipitated with ethanol and the precipitations were collected and suspended in 50 μL of Tris‐EDTA buffer. DNA samples were stored at −80°C until processing.

The forward primer 319F (ACTCCTACGGGAGGCAGCAG) and reverse primer 806R (GGACTACHVGGGTWTCTAAT) were used to amplify the V3‐V4 regions of the bacterial 16S rRNA gene (95°C for 2 min, followed by 25 cycles at 95°C for 30 s, 55°C for 30 s, 72°C for 30 s and a final extension at 72°C for 5 min). The amplicons, which were extracted from 2% agarose gels, were purified using an Axyprep DNA Gel Extraction Kit (Axygen Biosciences, Union City, CA, USA) according to the recommended instructions and quantified using Quantifluor™‐ST (Promega, Madison, Wisconsin, USA). The purified amplicons were pair‐end sequenced on an Illumina MiSeq platform according to a standard protocol (Caporaso *et al*., 2012). The barcodes unique to each sample were used to differentiate the different samples.

Raw FASTQ files were de‐multiplexed and quality‐filtered using QIIME (version 1.70, Flagstaff, Arizona, USA) with standard criteria as described previously (Mao *et al*., 2015). OTUs were clustered with a 97% similarity cut‐off using UPARSE (version 7.1 http://drive5.com/uparse/), and chimeric sequences were identified and removed using UCHIME (Edgar, 2010). The most abundant sequences within each OTU were designated as representative sequences and were classified using the Ribosomal Database Project classifier with a standard minimum support threshold of 80% (Wang *et al*., 2007). The microbial community diversity was estimated using the observed species, Simpson, Chao 1 and Shannon indices. An unweighted distance‐based analysis of molecular variance was conducted to assess significant differences among samples using the program MOTHUR v.1.29.0 (Schloss *et al*., 2009).

### Predicted molecular functions based on 16S rRNA data using PICRUSt

The present study used PICRUSt to predict the molecular functions of each sample based on 16S rRNA data. PICRUSt is a bioinformatics tool that uses marker genes, in this case 16S rRNA, to predict the gene functional content of microorganisms. These predictions are pre‐calculated for genes in various databases, including KEGG and COGs. The present study used the KEGG database and performed closed reference OTU picking using the sampled reads against a Greengenes reference taxonomy (Greengenes 13.5), using the pick_closed_reference_OTU.py script in QIIME. Within the online version of PICRUSt, the 16S copy number was normalized using the normalize_by_copy_number.py script, and molecular functions were predicted using the predict_metagenomes.py and data were summarized into KEGG pathways using the categorize_by_function.py script. In addition, PICRUSt calculated the NSTI to quantify dissimilarity between reference genomes and the predicted metagenome presented here.

### Quantification of the total bacteria by q‐PCR and gene expression analysis

The frozen colonic mucosa was homogenized in 1 ml TRIzol Reagent (Invitrogen, Carlsbad, CA, USA) and total RNA was isolated according to the manufacturer's recommendations. The total RNA was reverse‐transcribed to cDNA using a PrimeScript RT reagent Kit with gDNA eraser (TaKaRa Biotechnology, Dalian, China) according to the recommended procedures. The quantitative PCR was carried out on an Applied Biosystems 7300 Real‐Time PCR system (Foster City, CA, USA) using a SYBR Premix Ex Tag™ (Tli RnaseH Plus) qPCR kit (TaKaRa Biotechnology (Dalian), Dalian, China) according to the manufacturer's guidelines. The cycling conditions were 95°C × 30 s, followed by 40 cycles of 95°C × 5 s and 60°C × 34 s. The primers used are listed in Table S3. The expression of target genes relative to a housekeeping gene (â‐actin) was calculated using the 2^−ΔΔCt^ method (Livak and Schmittgen, 2001). The relative mRNA expression of the target gene was normalized to the control group. The gene copies of total bacteria in the colonic content were quantified by the standard curve method using q‐PCR (Rinttilä *et al*., 2004).

### Statistical analysis

Data were analysed using SPSS 16.0 (IBM, New York, USA) and are expressed as the means ± SEM. Both parametric (*t*‐test and ANOVA) and nonparametric methods (Kruskal–Wallis test and Mann–Whitney *U*‐test) were used to assess the differences between treatments. The normality of the distribution of variables was tested by the Shapiro–Wilk test. Parametric tests were used to analyse the variables with a normal distribution. The variables that had a non‐normal distribution were analysed using the nonparametric methods. The *t*‐test and the Mann–Whitney *U*‐test were used to analyse the data that had a normal or non‐normal distribution on PWD 14 respectively. A one‐way ANOVA with LSD post‐hoc comparison procedure was used to analyse the data with a normal distribution on PWD 28 and PWD 70. In addition, the data that had a non‐normal distribution on PWD 28 and PWD 70 were analysed by the Kruskal–Wallis test followed by the Mann–Whitney *U*‐test according to Etxeberria *et al*. (2015). The Mann–Whitney *U*‐test was used to compare the differences between two groups. Differences were considered significant when *P* < 0.05.

## Supporting information


**Fig. S1.** Rarefaction curves of Chao 1, Shannon, Simpson and species accumulation curve.C1 represents sampleson PWD 14; C2 represents sampleson PWD 28; C3 represents sampleson PWD 70.
**Fig. S2.** The average relative abundance of predominant bacteria at the phylum level in the colonic content on PWD 14, PWD 28 and PWD 70.
**Fig. S3.** The average relative abundance of predominant bacteria at the family level in the colonic content on PWD 14, PWD 28 and PWD 70.
**Fig. S4.** The average relative abundance of predominant bacteria at the genus level in the colonic content on PWD 14, PWD 28 and PWD 70.
**Fig. S5.** Comparisons of the average relative abundance of altered OTUs on PWD 14, PWD 28 and PWD 70.Data were represented as the mean±SEM. * indicates *p* < 0.05; ** indicates *p* < 0.01.
**Fig. S6.** Metagenomic functions of the bacteria in the colonic content presented as the relative abundances of KEGG pathways.
**Table S1.** The alpha diversity as indicated by OTUs, Shannon, Simpson and Chao 1 indices.
**Table S2.** Diet^1^ formula used in this study.
**Table S3.** Primers used in this study.
