# Supplementary material for: The role of microbiota in compensatory growth of protein‐restricted rats
Source: Microb Biotechnol. 2016 Nov 22;10(2):480–91. doi: 10.1111/1751-7915.12451 (PMC5328828; doi:10.1111/1751-7915.12451)
Supplement: Supplementary file 1 — Fig. S1. Rarefaction curves of Chao 1, Shannon, Simpson and species accumulation curve.C1 represents sampleson PWD 14; C2 represents sampleson PWD 28; C3 represents sampleson PWD 70. Fig. S2. The average relative abundance of predominant bacteria at the phylum level in the colonic content on PWD 14, PWD 28 and PWD 70. Fig. S3. The average relative abundance of predominant bacteria at the family level in the colonic content on PWD 14, PWD 28 and PWD 70. Fig. S4. The average relative abundance of predominant bacteria at the genus level in the colonic content on PWD 14, PWD 28 and PWD 70. Fig. S5. Comparisons of the average relative abundance of altered OTUs on PWD 14, PWD 28 and PWD 70.Data were represented as the mean±SEM. * indicates p < 0.05; ** indicates p < 0.01. Fig. S6. Metagenomic functions of the bacteria in the colonic content presented as the relative abundances of KEGG pathways. Table S1. The alpha diversity as indicated by OTUs, Shannon, Simpson and Chao 1 indices. Table S2. Diet1 formula used in this study. Table S3. Primers used in this study. [file MBT2-10-480-s001.docx]

**Supporting information**

**Figure S1.** Rarefaction curves of Chao 1, Shannon, Simpson and species accumulation curve. C1, C2 and C3 represent PWD 14, PWD 28 and PWD 70, respectively. According to **Table 3**, the least average number of sequenced reads was 19391.33. At this sequencing depth, all the rarefaction curves and species accumulation curve reached a plateau, indicating that the sampling was sufficient to evaluate the bacterial community profile in the colonic content.

**Figure S2.** The average relative abundance of predominant bacteria at the phylum level in the colonic content on PWD 14, PWD 28 and PWD 70. The most dominant bacteria was Firmicutes, accounting for 72.90 - 94.18 % of the total sequences, followed by the bacteria from the phyla Bacteroidetes (2.58 - 23.54 %), Actinobacteria (0.86 - 5.04 %), Candidatus Saccharibacteria (0.12 - 1.36 %), and Proteobacteria (0.25 - 0.92 %).

**Figure S3.** The average relative abundance of predominant bacteria at the family level in the colonic content on PWD 14, PWD 28 and PWD 70. At the family level, 52 families were detected, including 6 unclassified ones. The bacterial taxa that had an average relative abundance of > 1 % in at least one group, were statistically analyzed. As a result, 16 families of bacteria were asessed, with Lactobacillaceae the most predominant (28.40 - 58.70 %).

**Figure S4.** The average relative abundance of predominant bacteria at the genus level in the colonic content on PWD 14, PWD 28 and PWD 70. At the genus level, 103 genera were detected, including 23 unclassified genera. The genera that had a relative abundance of > 2 % in at least one group were analyzed. As a result, 13 genera of bacteria, including the unclassified Porphyromonadaceae, Lachnospiraceae, Peptostreptococcaceae, and Ruminococcaceae were analyzed. The most dominant genus was *Lactobacillus* (28.40 - 58.70%), followed by unclassified Lachnospiraceae (6.98 - 33.66%), and unclassified Peptostreptococcaceae (3.22 - 26.03%).

**Figure S5.** Comparisons of the average relative abundance of altered OTUs on PWD 14, PWD 28 and PWD 70. Data were represented as the mean±SEM. * indicates p < 0.05; ** indicates p < 0.01. The OTU 3557, belonging to Peptostreptococcaceae, was enriched in the L group on PWD 14, PWD 28 and PWD 70 compared with the N group. Moreover, the abundance of OTU 3557 was increased in the LN group on PWD 28 and PWD 70. Similarly, the OTU 3407, which belongs to *Ruminococcus*, was also enriched in the L group on PWD 14 and PWD 28 and an enriched abundance of OTU 3407 was observed on PWD 28 in the LN group. However, the abundance of OTU 1960 (belonging to *Prevotella*), OTU 1898 (belonging to *family S24-7*) and OTU 1802 (belonging to *Bacteroides*) was decreased in the L group on PWD 14, PWD 28 and PWD 70, and was decreased in the LN group on PWD 28 and PWD 70.

**Figure S6.** Metagenomic functions of the bacteria in the colonic content presented as the relative abundances of KEGG pathways. 37 KEGG pathways were identified in the colonic content. Among the 37 genes indentified, the top 5 major KEGG pathways are membrane transport (14.39 - 32.43 %), carbohydrate metabolism (10.87 - 16.83 %), amino acid metabolism (4.39 - 8.84 %), replication and repair (2.86 - 8.23 %), and energy metabolism (4.02 - 6.09 %).

**Table S1.** The alpha diversity as indicated by OTUs, Shannon, Simpson and Chao 1 indices. The bacterial diversity in the colonic content of rats in the L group decreased on PWD 28. For the colonic bacterial diversity of rats in the LN group, the values of observed OTUs, Shannon, Simpson and Chao 1 indices did not differ from those in the N group on PWD 28 and PWD 70, which indicated that protein restriction for 14 days did not affect the bacterial diversity in the colonic content of rats in the LN group.

**Table S2.** Diet**^1^** formula used in this study. The normal protein diet (NPD) was formulated according to the AIN-93G with a 19.34 % of crude protein. The low protein diet (LPD) was designed with a 12 % of crude protein.

**Table S3.** Primers used in this study. The primer sequences were either acquired from published literatures or designed by using the online version of picking-primers in NCBI in this study.


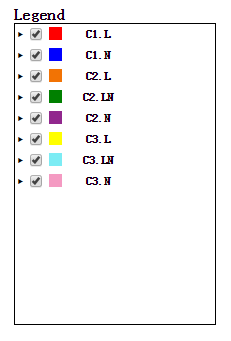

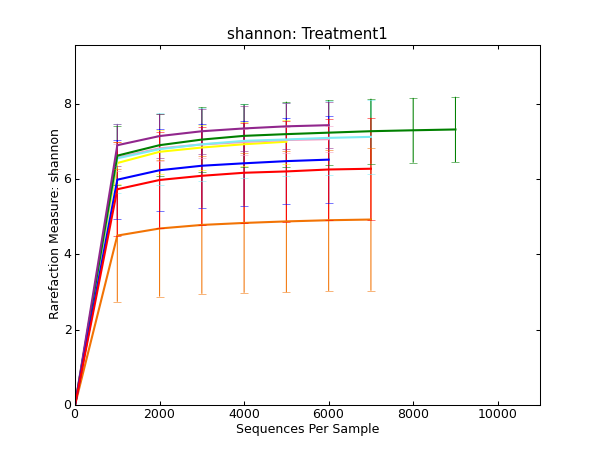

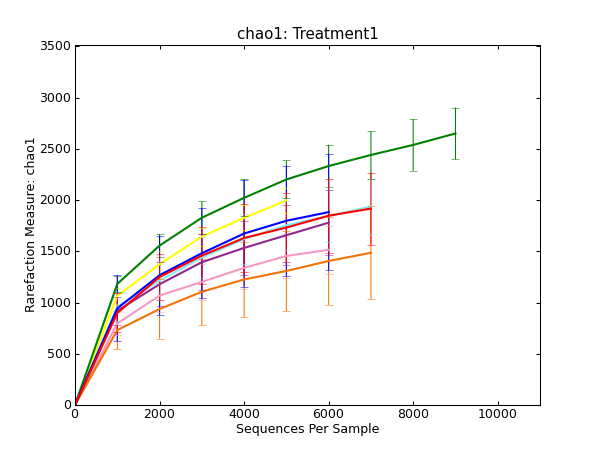


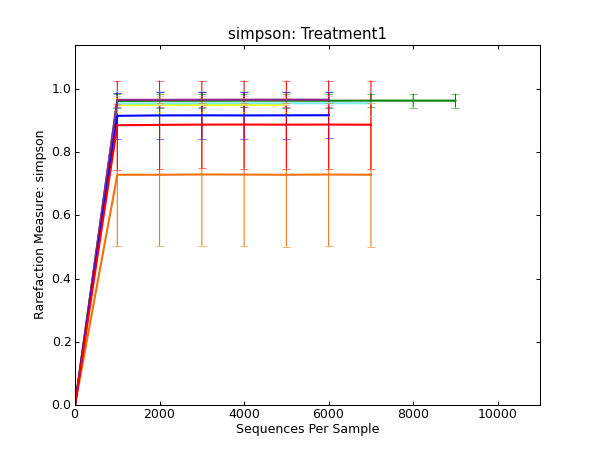


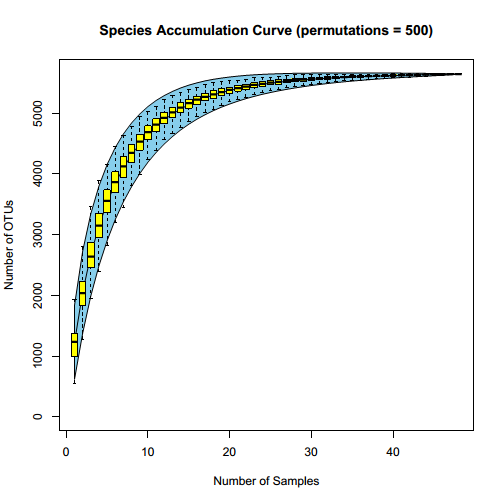


**Fig S1.** Rarefaction curves of Chao 1, Shannon, Simpson and species accumulation curve. C1 represents sampleson PWD 14; C2 represents sampleson PWD 28; C3 represents sampleson PWD 70.


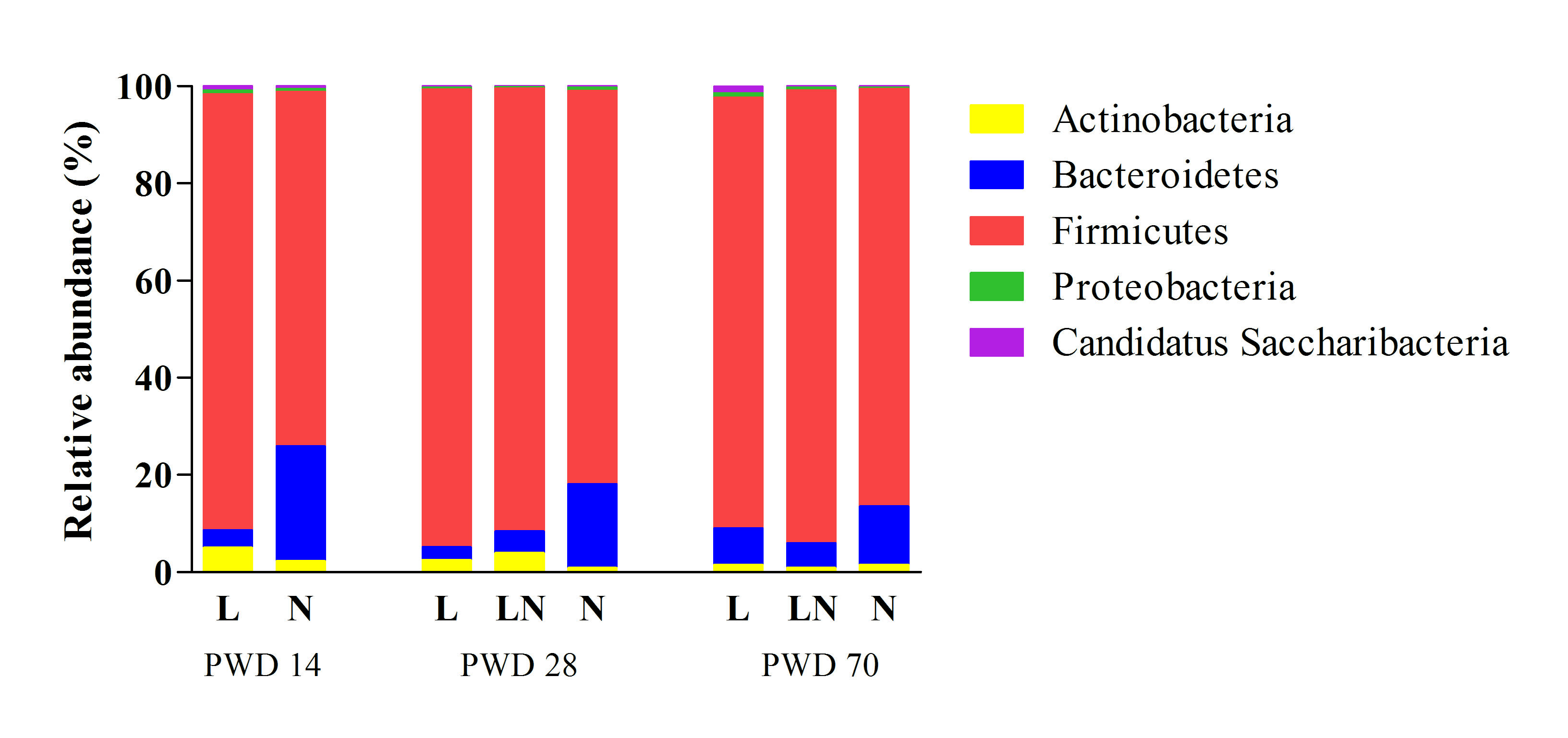


**Fig S2.** The average relative abundance of the predominant bacteria at the phylum level in the colonic contenton PWD 14, PWD 28 and PWD 70.


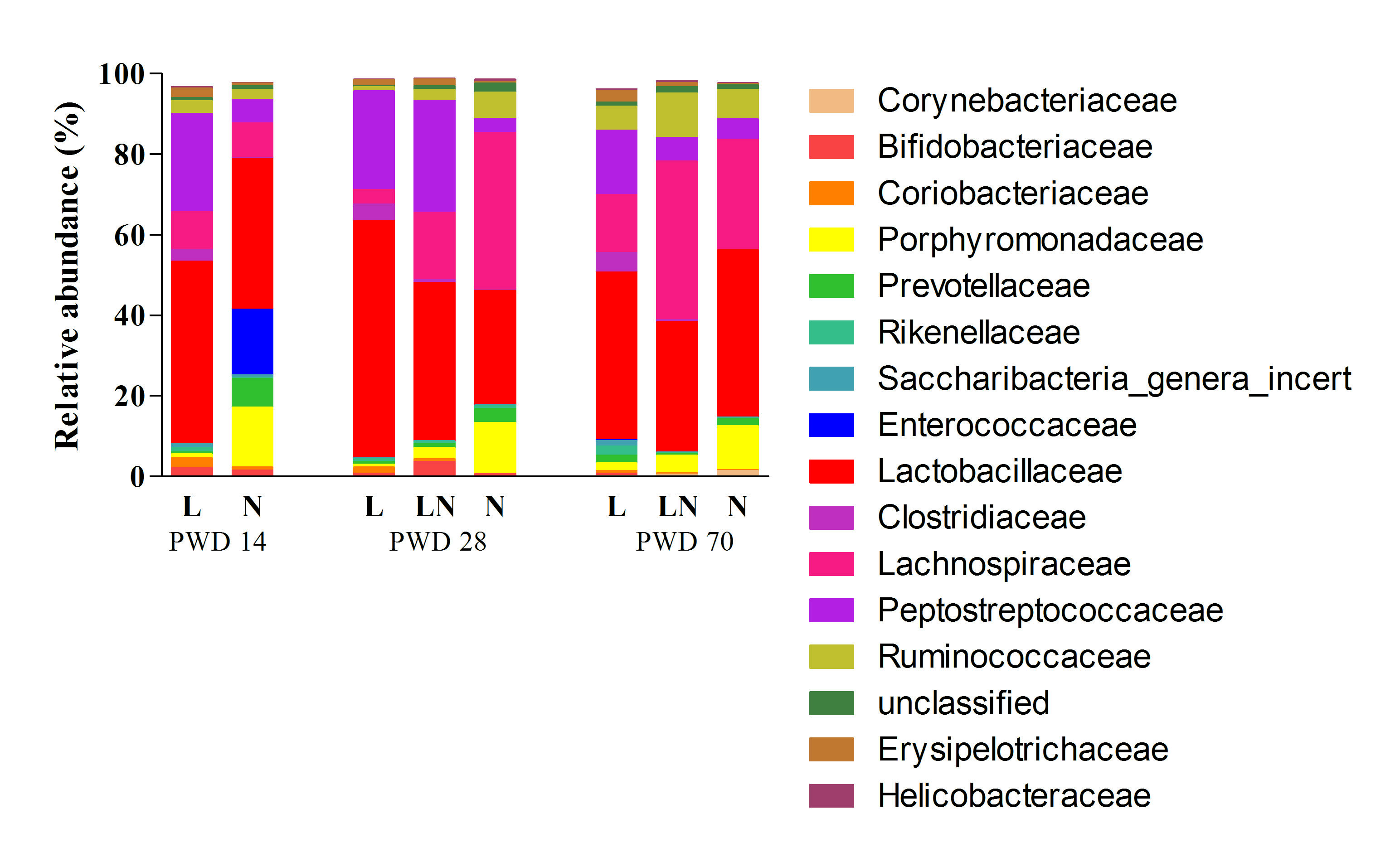


**Fig S3.** The average relative abundances of predominant bacteria at the family level in the colonic content on PWD 14, PWD 28 and PWD 70.


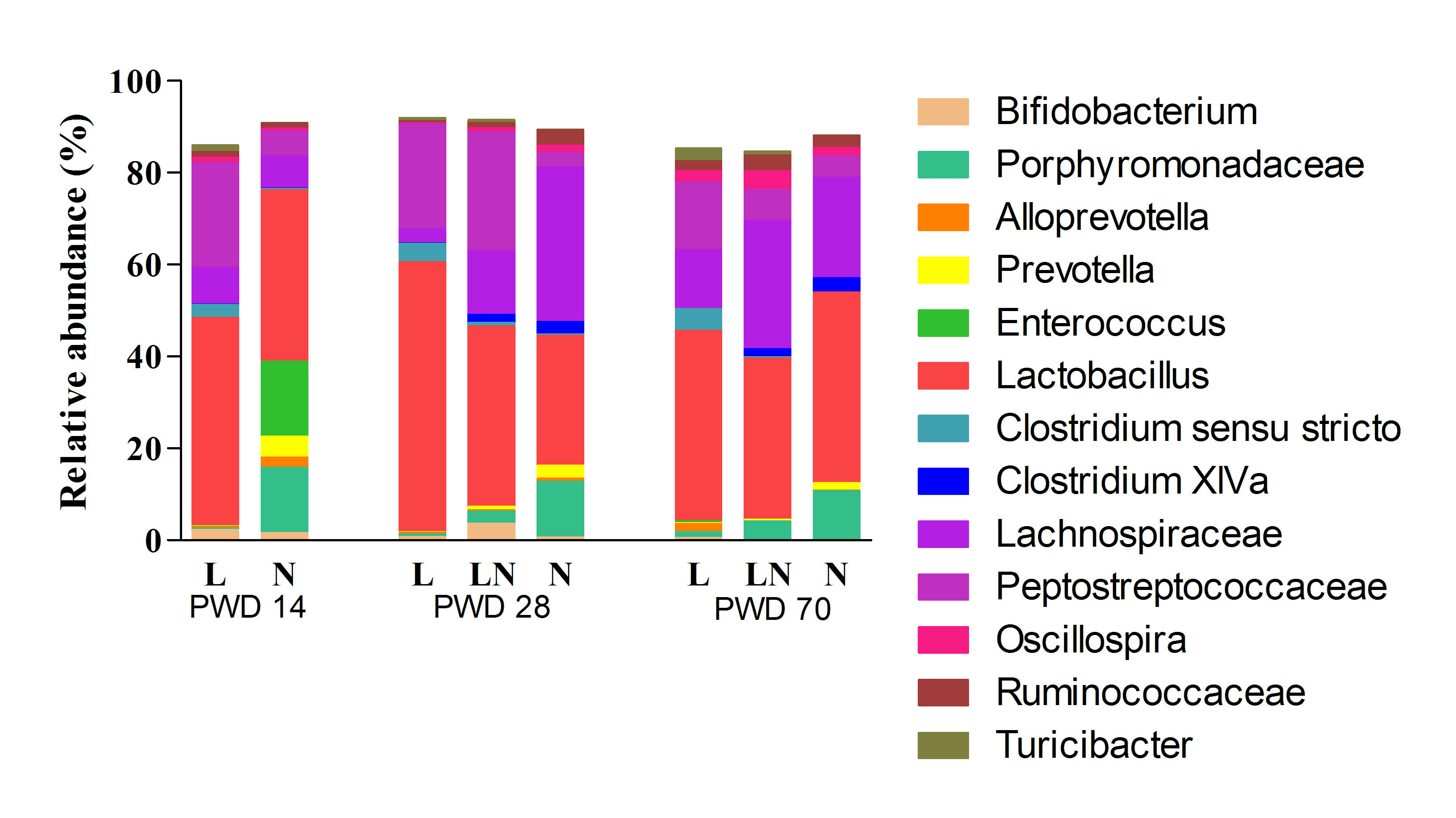


**Fig S4.** The average relative abundances of predominant bacteria at the genus level in the colonic content on PWD 14, PWD 28 and PWD 70.


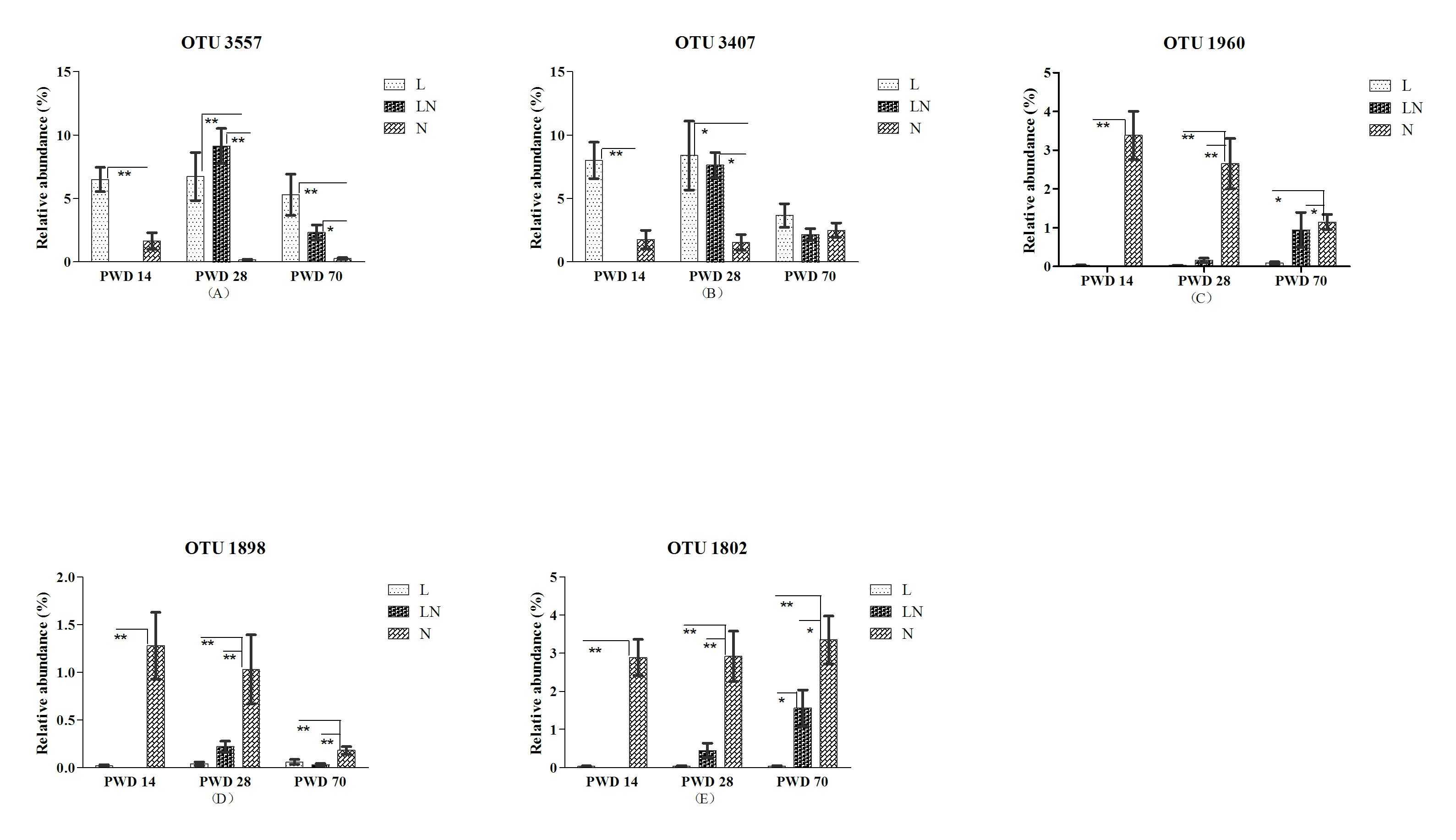


**Fig S5.** Comparisons of the average relative abundances of altered OTUson PWD 14, PWD 28 and PWD 70. Data were represented as the mean±SEM. * indicates *p* < 0.05; ** indicates *p* < 0.01.

**Fig S6.** Metagenomic functions of the bacteria in the colonic content presented as the relative abundances of KEGG pathways.

**Table S1.** The alpha diversity as indicated by OTUs, Shannon, Simpson and Chao 1 indices.

|  | PWD 14 | | |  | PWD 28 | | | |  | PWD 70 | | | |
| --- | --- | --- | --- | --- | --- | --- | --- | --- | --- | --- | --- | --- | --- |
|  | L | N | SEM |  | L | LN | N | SEM |  | L | LN | N | SEM |
| OTUs | 878.60 | 938.80 | 89.26 |  | 643.20^a^ | 1032.00^b^ | 960.00^b^ | 52.27 |  | 1052.00^a^ | 954.20^ab^ | 837.25^b^ | 41.22 |
| Shannon | 6.11 | 6.38 | 0.55 |  | 4.39^a^ | 6.80^b^ | 7.66^b^ | 0.36 |  | 7.17 | 7.26 | 7.15 | 0.22 |
| Simpson | 0.87 | 0.91 | 197.76 |  | 0.68^a^ | 0.95^b^ | 0.98^b^ | 0.03 |  | 0.96 | 0.96 | 0.97 | 0.01 |
| Chao 1 | 1766.88 | 2010.76 | 0.05 |  | 1222.76^a^ | 2160.20^b^ | 1681.39^c^ | 103.54 |  | 2028.41^a^ | 1711.50^ab^ | 1429.69^b^ | 73.20 |

Means in the same row with different superscripts in a colum represent a significant difference ( *p* < 0.05)

**Table S2.** Diet**^1^** formula used in this study.

| Ingredients（g/100g） | NPD | LPD |
| --- | --- | --- |
| corn | 49.15 | 61.15 |
| wheat | 4.45 | 15.00 |
| cornstarch | 5.91 | 5.00 |
| soybean meal | 27.99 | 4.25 |
| fish meal | 2.00 | 2.00 |
| soybean oil | 5.00 | 5.00 |
| L-lysine | 0.45 | 1.13 |
| DL-methionine | 0.37 | 0.48 |
| L-threonine | 0.10 | 0.31 |
| limestone | 2.61 | 3.00 |
| calcium hydrogen phosphate | 1.40 | 2.15 |
| salt | 0.20 | 0.20 |
| vitamin mix**^2^** | 0.11 | 0.10 |
| mineral mix**^3^** | 0.25 | 0.24 |
| total | 100.00 | 100.00 |
| Nutritional value |  |  |
| digestible energy (Mcal/kg) | 3.40 | 3.40 |
| crude protein % | 19.34 | 12.00 |
| Lys % | 1.37 | 1.30 |
| Met % | 0.68 | 0.69 |
| (Met + Cys) % | 0.96 | 0.83 |
| Thr % | 0.84 | 0.70 |
| apparent P % | 0.40 | 0.51 |
| total P % | 0.61 | 0.68 |
| Ca % | 1.42 | 1.68 |
| DM % | 89.10 | 89.33 |

**^1^**formulated according to the AIN-93G;

**^2^**formulated according to the Vitamin Mix V10001;

**^3^**formulated according to the Mineral Mix S10026.

**Table S3.** Primers used in this study.

| Gene name | | accession number | | primer sequences | | reference | |
| --- | --- | --- | --- | --- | --- | --- | --- |
| β-actin | | NM_031144.3 | | sense-CACGATGGAGGGGCCGGACTCATC | | ([Zhou and Zhou, 2011](#_ENREF_3)) | |
|  |  |  |  | antisense-TAAAGACCTCTATGCCAACACAGT | |  |  |
| mucin-1 | | NM_012602.1 | | sense-CAGTGCCGCCGAAAGAGC | | ([Lin et al., 2002](#_ENREF_1)) | |
|  |  |  |  | antisense-CTAACGTGGTGACAAAGC | |  |  |
| mucin-2 | | NM_023566.3 | | sense-AAGCCAGATCCCGAAACCAT | | ([Lin et al., 2002](#_ENREF_1)) | |
|  |  |  |  | antisense-ATGGCCCCATTCACAACTGCC | |  |  |
| mucin-3 | | XM_008760848.1 | | sense-GGTACAGCGGTGAAAACT | | ([Lin et al., 2002](#_ENREF_1)) | |
|  |  |  |  | antisense-CATGGGGAAATCTCAACG | |  |  |
| mucin-4 | | XM_006248471.2 | | sense-GCTTGGACATTTGGTGATCC | | ([Paturi et al., 2013](#_ENREF_2)) | |
|  |  |  |  | antisense-GCCCGTTGAAGGTGTATTTG | |  |  |
| TLR 1 | | NM_001172120.2 | | sense-TGCTTGAGGACAAGAACTGC | | designed in this study | |
|  |  |  |  | antisense-TTCCATGTTGTGTCCACGTT | |  |  |
| TLR 2 | | NM_198769.2 | | sense-GAGGAAGCCCAAGAAAGCTC | |  |  |
|  |  |  |  | antisense-TGAAGGGTGGGTCAGAGTTC | |  |  |
| TLR 4 | | NM_019178.1 | | sense-GAGGACTGGGTGAGAAACGA | |  |  |
|  |  |  |  | antisense-AGATACACCAACGGCTCTGG | |  |  |
| IL-10 | | NM_012854.2 | | sense-GGAGTGAAGACCAGCAAAGG | |  |  |
|  |  |  |  | antisense-GGCAACCCAAGTAACCCTTA | |  |  |
| TGF-β | | NM_021578.2 | | sense-GTCAACTGTGGAGCAACACG | |  |  |
|  |  |  |  | antisense-AGACAGCCACTCAGGCGTAT | |  |  |
| Occludin | | NM_001106266.1 | | sense-CCTTACAGGCCGGATGAAT | |  |  |
|  |  |  |  | antisense-GCTCTGTCCCAAGCAAGTGT | |  |  |
| ZO-1 | | NM_031329.2 | | sense-CCTTTCGCCTGAAACAAACC | |  |  |
|  |  |  |  | antisense-CTACATGCGACGGCAATGAC | |  |  |

**References**

Lin, J., Tsuboi, Y., Pan, W., Giebink, GS., Adams, GL., Kim, Y. (2002) Analysis by cDNA microarrays of altered gene expression in middle ears of rats following pneumococcal infection. *Int J Pediatr Otorhinolaryngol* **65**:203-211.

Paturi, G., Bentley‐Hewitt, KL., Butts, CA., Nyanhanda, T., Monro, JA., Ansell, J. (2013) Dietary combination of potato resistant starch and red meat up‐regulates genes involved in colonic barrier function of rats. *Int J Food Sci Technol* **48**:2441-2446.

Zhou, J-Y., Zhou, S-W. (2011) Protective effect of berberine on antioxidant enzymes and positive transcription elongation factor b expression in diabetic rat liver. *Fitoterapia* **82**:184-189.
